# Clinicians’ Perceptions of Norwegian Women’s Experiences of Infertility Diseases

**DOI:** 10.3390/ijerph17030993

**Published:** 2020-02-05

**Authors:** Alexandra Fernandes, Lotte-Lise Skotnes, Maria Major, Pedro Fontes Falcão

**Affiliations:** Business Research Unit (BRU-IUL), Instituto Universitário de Lisboa (ISCTE-IUL), Avenida das Forças Armadas, 1649-026 Lisboa, Portugal; lotteskotnes@gmail.com (L.-L.S.); maria.joao.major@iscte-iul.pt (M.M.); pfontesfalcao@iscte-iul.pt (P.F.F.)

**Keywords:** women’s health, women’s chronic diseases, Norway, public health, infertility, gynecology, birth rate improvement

## Abstract

Background: Norway has one of the best health systems in the world. However, it has a low birth rate, which decreased by 21.2% between 2009 and 2018, and one of the highest rates of infertility prevalence. The aim of this study is to understand how Norwegian doctors perceive female infertility diseases, namely those that are more difficult to diagnose and to treat, and that are more common in their practice. Method: Descriptive qualitative study was conducted with gynecologists and general practitioners. The sample resulted from the establishment of five criteria and on the doctors’ acceptance to participate in this study. Our sample comprised thirteen highly qualified and experienced doctors. Qualitative content analysis was the method chosen to analyze the collected data. Results: Clinical diseases (polycystic ovary syndrome, endometriosis and vulvodynia) and consequences of these diseases were the pinpointed themes. These led to a set of sub-themes: the main symptoms and the treatment of the diseases, from the perspective of both women and doctors (stigmatization, disturbances in women’s daily life, diagnostic delay, and governmental support). Conclusions: The three most relevant disorders mentioned were polycystic ovary syndrome, endometriosis and vulvodynia. These diseases cause several impacts on the lives of women, because they feel stigmatized and limited in their daily life and sexuality, and the diagnosis of these diseases takes too much time. Governments should better redistribute the financing of women’s health and allocate resources to specialized centers.

## 1. Introduction

Over the last few decades new priorities in public health have led to improvements on the study of women’s health, enlarging the approach to include sexual and reproductive health. Moreover, in OECD countries infertility has become an important issue and reproductive health has started to be addressed as a priority, particularly due to the demographic ageing and low levels of natality [1]. However, infertility is a complex issue to deal with and to understand [2]. It is defined as “the failure to achieve a clinical pregnancy after 12 months or more of regular unprotected sexual intercourse” [3] (p. 2686). A major study conducted by the World Health Organization (WHO), comprising 8500 couples from 25 countries, concluded that the specific causes of infertility in women were mainly due to ovulatory disorders, endometriosis, pelvic adhesions, tubal blockage, other abnormalities, and hyperprolactinemia [4]. Some of these problems were amplified by the continuous postponing of the average age of pregnancy, and also by some changes in women sexual behavior (e.g., number of sex partners, higher incidence of sexually transmitted diseases) [5]. Increasing incidences of diabetes, hypertension, hypothyroidism and lifestyle diseases also have negative consequences in female fertility [6], not to mention the growth of systemic autoimmune diseases [7]. Despite these improvements on the comprehension of infertility, its prevalence, and treatment seeking, much more research is necessary [8]. For instance, it is not yet understood how the conjunction of genes with fluctuating female sex hormones affect several bodily functions, including reproductive ones [9]. Likewise, infertility issues seem to vary according to contexts, thus it needs to be addressed according to regional, cultural and demographic specificities [6,10]. Finally, infertility studies must also address the psychological and social side-effects of this problem, namely how it affects women in particular [11,12,13].

Infertility is perceived by women as stigma and discrimination [14]. For many women, infertility can confront their core female identity, and a decreasing sense of self-worth can be created, not only because their body cannot function properly but also because their self-esteem has been injured [15]. However, among the variety of known infertility diseases it has not been clearly determined which ones cause more physical and psychological problems to women. This is particularly poignant among those that are most difficult to diagnose and treat, namely polycystic ovary syndrome [16], premenstrual dysphoric disorder [17], endometriosis [18], premature ovarian failure [19], vulvodynia [20], and chronic pelvic pain [21]. Our aim in this paper is to contribute to the literature on the physical and psychosocial consequences of infertility on women from the perspective of the clinicians who treat these women in a specific geographic context. In this case, we selected Norway to conduct this research, because it simultaneously has one of the best health systems in the world, one of the lowest birth rates and one of the highest rates of infertility prevalence.

According to the 2000 World Health Report produced by the World Health Organization (WHO), which analyzed the health systems of its 197 member states, Norway was ranked in the 3rd position [22]. In the 2018 Euro Health Consumer Index, presenting a comparison for national healthcare systems in 35 European countries, the Health System in Norway was also ranked in the 3rd position from the customer/consumer point of view [23]. In short, these indexes show that Norway is a country of reference in terms of health. At the same time, Norway has a major issue with low birth rates. Norwegian Prime Minister Erna Solberg has pointed out in several speeches that the country has a serious problem with the decreasing birth rate [24,25,26]. Although low birth rates cannot be overcome only by solving infertility problems, this can certainly contribute to mitigating that downward trend. The number of occurred births between 2008 and 2018 of women from age 15 to 49 has substantially declined. From 2009 to 2018, Norway’s birth rate decreased from 1.98 to 1.56, which corresponds to a decrease of 21.2% during that ten years [27]. In light of this reality, it is important to understand how Norway is dealing with infertility. In fact, infertility in Scandinavian countries has an estimated median prevalence of 10–20% [28], compared to an estimated median prevalence of 9% worldwide [8]. Therefore, the urgency of addressing this topic is evident. This gap in knowledge motivated us to investigate how Gynecologists and General Practitioners (GP) in Norway perceive: (i) female infertility diseases in their practice; (ii) how they deal with these diseases; (iii) how they explain the consequences of these diseases to their female patients.

## 2. Materials and Method

### 2.1. Design

The main goal of this paper is to comprehend Norwegian physicians’ perceptions of female infertility diseases, namely those that are more difficult to diagnose and to treat, and that are more common in their practice. To uncover the perceptions of GPs in Norway towards infertility and the perceived consequences of gynecological diseases the most adequate research approach consists of qualitative studies [29]. Qualitative research has been recommended to produce in-depth explanations about phenomena within the context they operate [30]. Patton [31] adds that qualitative studies allow understanding social contexts in a holistic way and to get explanations for the ‘why’ and ‘how’ questions. Moreover, Greil [32] conducted a study about infertility stating that “descriptive and qualitative research should not be dismissed as it sometimes is as being merely “anecdotal” (p. 1700). The adoption of qualitative methods is regarded as particularly useful with respect to the study of the perception of infertility, with several studies taking the approach [33,34,35]. Our qualitative study was conducted between July and September of 2019 and was based on semi-structured in-depth interviews with GPs and gynecologists.

### 2.2. Study Design, Participant Selection, and Data Collection

The inclusion criteria to select the participants in this study were: (a) being gynecologists or GP with expertise in the field of women’s reproductive health; (b) having the same proportion of gynecologists and GP; (c) having an equal proportion of male and female doctors; (d) coming from Norway’s main cities; (e) having a higher academic education, namely, PhD. To find physicians with those characteristics, author A searched through Google in Norwegian hospitals, private practices, universities and published articles. It was possible to find a sample composed of 35 doctors from Oslo, Stavanger, Bergen, Trondheim, and Tromsø. These clinicians were invited to participate in this research by email, explaining that the aim of this study was to comprehend the perception of Norwegian physicians about female infertility diseases, namely those that are more difficult to diagnose and to treat, and that are more common in their practice. It was explained that their participation in the research consisted of an interview that could be done by telephone or face-to-face. Guarantees were given regarding anonymity of the collected data. A sample of 13 physicians accepted to participate in this study. Ten doctors came from Oslo, one doctor came from Bergen, one doctor came from Trondheim and one doctor came from Tromsø.

According to Table 1, it is possible to observe that 13 interviews were conducted with eight gynecologists and five GPs, and that ten of them had a PhD, which represents 76.9% of the sample. Despite an equal proportion of male and female doctors being contacted, the results were unbalanced: ten female and three male doctors responded (78% against 22%). However, this imbalance mirrors the much higher number of female doctors working in the gynecological area, and therefore it is more representative of the existing proportion between male and female doctors working in Norway. The interviews were mainly conducted by phone, except for two face-to-face interviews. These interviews lasted from 30 min to 110 min, with an average of 43 min per interview. Doctors’ professional experience ranged from 9 to 45 years, with an average of 30 years. More than 61.5% of the physicians had more than 30 years of experience (see Table 2). According to Morse [36], data saturation is a problem when qualitative samples are relatively small, so the sample “must be adequate (large enough for replication to occur and be noted) and appropriate (those interviewed must be experts in the phenomenon of interest)” (p. 588). Data saturation was achieved in this study because it involved a considerable number of interviewees who were specialists in Norway, and who were carefully identified as experts in women’s diseases, thereby allowing the triangulation of evidence and the confirmation of findings.

The aim of the study was explained at the beginning of the interviews. The example was given of diseases that were more often pinpointed in the specialized literature: polycystic ovary syndrome, premenstrual dysphoric disorder, endometriosis, premature ovarian failure, vulvodynia, and chronic pelvic pain. Then, participants were invited to express their view on how these diseases affect women, and which were the most common ones according to their professional experience.

Based on the literature review, Table 3 presents two thematic sections: (1) clinical diseases and (2) consequences of diseases, and the main questions of the interview guide.

### 2.3. Data Analysis

Qualitative content analysis was the method chosen for analyzing data. In qualitative data the difficulty is to assure its reliability and validity [37]. To overcome this, the rigor was shown by an accurate representation [38]. To ensure the quality of data, all interviews were audio-recorded with the permission of the participants and sent to them to ensure that they were transcribed correctly and to give them the opportunity to remove errors or imprecise statements.

The software Atlas.ti version 8 was used, which allowed two researchers to work simultaneously through a large quantity of text summarizing the content into smaller conjuncts and enabling the comprehension of the investigated phenomena [39]. Hence, the transcribed interviews were read several times by two authors to identify the codes connected with subthemes that showed up from the direct questions or somewhere in the interview. It was identified that in clinical diseases the participants described each disorder specifically, but in the consequences of the diseases almost all answers were given as global descriptions without pinpointing a specific disorder. Because a qualitative research design was adopted, the researchers were not interested in generating statistical generalizations, but in-depth explanations as to how doctors perceive gynecological diseases and infertility in Norway. Such explanations, if replicated in other studies, would allow the production of analytical generalizations [30].

### 2.4. Ethics

The purpose and nature of the study was disclosed to all participants during the introduction to the interviews, and informed consent was obtained. Participants were informed that at any time they could withdraw from the study without giving any reason. All interviews were audio-recorded for later transcription and the interviews were sent to the participants to confirm that their interviews were correctly transcribed.

The study was conducted by pursing the ethical principles of the Declaration of Helsinki. Personal data was presented rendering the Regulation (EU) 2016/2017 of European Parliament and in the Council of 27 April 2016 on the protection regarding personal data.

## 3. Results

**Figure 1:**
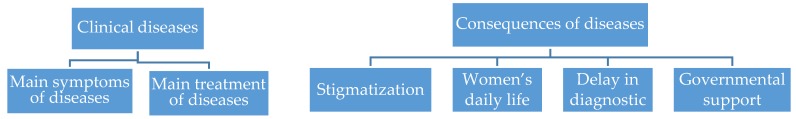
Themes and sub-themes. Source: prepared by the authors.

When asked which were the female infertility diseases that were more difficult to diagnose and to treat, and which were more common in their practice, almost every doctor said that polycystic ovary syndrome (PCOS), endometriosis, and vulvodynia were the most representative ones. The application of Atlas.ti version 8 software allowed the researchers to deepen the analysis and to synthetize the following themes, subthemes and codes (Table 4):

Figure 1 synthetizes this table’s data:

### 3.1. Main Symptoms of Diseases

Participants mentioned that PCOS is a disease that occurs because women have a hormonal disorder in their reproductive age:

*“* *PCOS includes problems with metabolism, obesity, hirsutism, acne and pregnancy. A large number of women with PCOS struggle to become pregnant.”*

*—G13*

Participants pointed out that endometriosis have some specific symptoms, but it is difficult to diagnose. It involves extreme pain during menstruation, and is defined by the endometrial tissue growing outside of the uterus which is difficult to remove:

*“Endometriosis is a kind of disease whose symptoms are painful periods and painful intercourse, with excessive bleeding. It takes some time until it is diagnosed. The only way to identify it is through a very invasive diagnostic called the laparoscopy method.”*

*—G1*

*“Endometriosis involves painful periods. But in more serious cases, the tissue can travel to the intestines. I even had one patient where the endometriosis had travelled to the lungs, which is a more rare and extreme case.”*

*—G13*

*“They call it the mushroom disease because it keeps coming back after surgery.*

*—G1*

Participants pointed out that vulvodynia is difficult to diagnose. Women with vulvodynia complained that they feel a burning pain or discomfort around their vulva followed by muscle cramps and tightening (vaginism):

*“Vulvodynia has been a problem. We don’t know the causes or how to really treat it, but it is a big problem. Women suffer a lot. Vulvodynia has been related to stress, the use of contraceptive pill, maybe over the counter ointments for suspected Candida and get hypersensitivity in this area.”*

*—G1*

*“The worst consequence of vulvodynia is that women have tightening muscles, negative effects on sexual health and pain. Vulvodynia patients are often around 20/25 years, which has quite dramatic consequences in their sexually active and reproductive years.”*

*—D8*

### 3.2. Main Treatment of Diseases

In terms of treatment for PCOS, participants pointed out that they use a combination of treatment, namely hormones/anti hormones, metformin, and surgery according to the patient situations:

*“One of the treatment methods for women with acne and PCOS, is to give them birth control pills.”*

*—G6*

*“With PCOS, ovulation is often missing, so they need general treatment and infertility treatment. (...) There have been discussions of the medication metformin as a treatment for these women to become pregnant more easily.”*

*—G7*

Participants pointed out that endometriosis can be treated based on hormonal therapy and surgery. The chosen approach will depend on how severe symptoms are:

*“The first treatment should be the contraceptive pill. This is the treatment that general practitioners could offer them without having the exact diagnosis, because the diagnosis is mostly only obtainable by laparoscopy. (…) If [it] doesn’t work we perform laparoscopy, to try to remove the disease.”*

*—G1*

“If [oral contraceptive without a break] does not work we perform laparoscopy, to try and remove the disease (endometriosis)”

Vulvodynia is a skin and muscle cramp (vaginism) condition that can be treated with medications and combined psychological interventions:

*“For vulvodynia there are four different treatment options. One is physiotherapy, medications, sexology, couples therapy, etc. And we also utilize self-training. Those are the four treatments we generally apply.”*

*—D8*

### 3.3. Stigmatization

Participants mentioned that many women who suffer from these diseases feel highly stigmatized:

*“You know, you need to spend time consulting the patient because these diseases are still a taboo and patients don’t speak about them easily.”*

*—D8*

*“When women seek help, then it is often because they have a real need. It is not always the case that they will need medications, but they should in any regard be taken seriously because they are not comfortable talking about those issues. (…) There is the stigma attached to reproductive health issues and women find it difficult to seek help.”*

*—D5*

### 3.4. Women’s Daily Life

Participants considered that infertility diseases seriously affect the normal life of women and can take a long time to be treated:

*“I deal with a lot of women who struggle with infertility. And it can become all-consuming in terms of psychosocial function and sexual health.”*

*—G3*

*“There are several studies demonstrating that women with endometriosis have a reduced quality of life. Unsurprisingly, many have chronic pain for 30–40 years.”*

*—G6*

*“It becomes difficult to participate socially, have a normal sex life or have the number of children you desire.”*

*—G10*

### 3.5. Delay in Diagnosis

Most participants pointed out that there is a delay in the diagnostic of these diseases, due to a combination of stigma and neglecting:

*“Additionally it is very taboo, the patients find it hard to talk about. And therefore I really feel they need to be seen.”*

*—D8*

*“Within women’s health many of the issues the women bring forward are not always defined under a specialty. Therefore few doctors take full responsibility for the diagnosis. (…) Women often have to fight hard for their diagnosis. They often have to seek several medical professionals and feel like it is a fight to be believed.”*

*—D5*

*“* *There is a problem with diagnostic delay in Norway. It can take 6–7 years from their first symptoms to occur, until diagnosis is reached. So this is a problem, because they will have many other diagnoses first, such as pelvic inflammatory diseases, bowel problems, urinary problems etc. Then they receive the wrong treatment in many cases.”*

*—G1*

### 3.6. Governmental Support

According to participants the amount of money that it is spent on maternal care is much higher than what is allocated to other dimensions of women’s health care:

*“Maternity care is wrongly financed, because departments are rewarded based on interventions and so forth.* *”*

*—G3*

*“* *Maternal care has been highly prioritized within gynecology, and there is nothing wrong with that. But I believe that more than 80% of the research at the women’s health center is on maternal care.”*

*—D8*

Participants point out that women suffer too much because it takes a long time until doctors recognize that they have a disease. There is a real need for having specialized centers with multidisciplinary knowledge to give support to these diseases:

*“Norway only has one large and one very small vulva specialized center in comparison to 20 centers each in Denmark and Sweden. The demand is extremely large and waiting lists are up to 6 months. (…) Nowadays there are endless waiting lists of doctors signing up for seminars about vulvodynia.”*

*—D8*

*“For example, vulva issues lie between gynecology, skin and infection, or neurology. Then there are many [areas] involved and it becomes highly fragmented.”*

*—D5*

## 4. Discussion

The diseases that are more difficult to diagnose and to treat, and that are more common in doctors’ practices, were identified by participants as PCOS, endometriosis, and vulvodynia. PCOS is a chronic disease associated with one or more of the following symptoms: insulin sensitivity, thyroid issues, hirsutism, infertility, cysts on the ovaries, acne and obesity, which affect the patient beyond the reproductive function [40]. Teede et al. [41] recommended pharmacological medication and surgical techniques to treat PCOS. Endometriosis is a chronic, estrogen-dependent, and inflammatory disease of unknown etiology and without a cure [18]. It is associated with endometrial tissue growing outside the uterus, severe pelvic pain, menstrual irregularities and infertility, leading to a significant reduction in the quality of life and sexual satisfaction [42]. The treatments usually imply hormonal medications or surgical intervention [42,43], although medical diagnosis depends on surgical visualization [44]. Finally, vulvodynia appears when women have a chronic vulvar “burning, stinging, rawness, soreness or pain” [45] (p. S24). Women have burning pain in their vulva, but it usually arises with other relevant clinically identifiable disorders such as neurological ones [20]. The diagnosis is based on the exclusion of other causes of pain [46]. According to Haefner et al. [47] vulvodynia treatment is based on medications, physical therapy and surgical techniques, although none of the participants mentioned surgical treatments as a solution.

Doctors pointed out that these diseases often imply embarrassment and stigma. Women avoid consulting a doctor because they feel guilt and discomfort for having to talk about sexual subjects with strangers [20]. This type of disease often requires intimate examination and a large amount of self-care; thus, if these obstacles remain, the development of women’s health can be hindered [48]. Furthermore, menstrual difficulties have been informed as one of women’s main self-reported health concerns, along with tiredness, overweight, depression, and anxiety [49]. These problems cause psychological morbidity and have a very negative impact on women’s daily life [50]. Women with chronic diseases feel “lack of comprehension, (…) rejected, ignored, and being belittled, blamed for their condition and assigned psychological explanation models” [51] (p. 1409). Indeed, “women patients exerted themselves to attract the doctor’s medical attention and interest and were anxious to be considered as whiners or complainers” [51] (p. 1409).

Although some articles have analyzed each disease individually and pointed out their consequences for women, as far as we were able to survey, no scientific research has previously studied together the three types of infertility discussed with the doctors (PCOS, endometriosis and vulvodynia) and their consequences for women’s daily life. Our investigation evidenced that those who suffer from PCOS have poor quality of life, due to physical and psychological problems associated with this disease, namely obesity, hirsutism, anxiety and depression [52]. Those who suffer from endometriosis have psychological problems such as anxiety, stress and depression, implying the loosing of working abilities and less social activities [53]. Finally, those who suffer from vulvodynia have high levels of anxiety, hopelessness and depression [20].

Still, it is very difficult to make a diagnostic of the diseases studied. Harlow [54] conducted a study and found that women experienced genital discomfort for three months or more. Specifically, women had to visit, on average, three doctors, often without resulting in a diagnosis. Another study showed that some of these diseases could take several years until being properly identified [55]. Finally, women can report so many complains that doctors can be confused and spend between 6 to 12 years to make a proper diagnosis [55,56,57].

Almost all doctors stated that in order to treat these categories of diseases it is important to have a multidisciplinary approach [58]. Agarwal [18] moved further and highlighted the need for establishing multidisciplinary centers that include mental health services with several specialties, namely gynecology, endocrinology, urology, surgery, psychology, and pain medicine. These centers allow patients to get a better treatment and can facilitate research and education.

## 5. Conclusions

This paper’s goal was to comprehend the perception of Norwegian physicians about female infertility diseases, namely those that are more difficult to diagnose and treat, and that are more common in their practice. According to our sample of Norwegian physicians, the most common disorders were PCOS, endometriosis and vulvodynia, which implied symptoms such as prolonged menstrual periods, excess of male hormones, endometrial tissues travel and big pain. With respect to the treatment of these diseases, physicians prescribed a combination of medications and surgical interventions. From this research, we were able to extract three main conclusions. Firstly, infertility diseases caused several limitations on the life of women, because they felt stigmatized and limited in their daily life and sexuality. Secondly, doctors work mainly by trial and error in the diagnostic of these diseases, implying too much time before a conclusive analysis is reached. Finally, governments should redistribute better the financing of women’s health and allocate resources to specialized centers to treat or manage these chronic diseases (PCOS, endometriosis and vulvodynia), and foster the diffusion of seminars and workshops about these diseases.

In terms of future research, it could be relevant to conduct more studies that: (i) include more clinicians of both sexes covering proportionally the main cities of Norway; (ii) replicate the study in the other three Scandinavian countries; (iii) build a questionnaire based on these studies that could be applied to other developed countries; (iv) analyze the impact on infertility and birth rates of those specialized centers and to compare them with other developed countries.

## Figures and Tables

**Table 1:** Characterization of the sample and duration of interviews.

ID. Code	Physician	Sex	PhD	Sector	Interview	Range (minutes)
G1	Gynecology	Female	X	Public	Phone	30–45
D2	GP	Female		Public	Face-to-face	>60
G3	Gynecology	Female	X	Public	Phone	45–60
G4	Gynecology	Female	X	Public	Face-to-face	30–45
D5	GP	Female	X	Public	Phone	45–60
G6	Gynecology	Male	X	Public	Phone	30–45
G7	Gynecology	Female	X	Public	Phone	30–45
D8	GP	Female	X	Public/Private	Phone	30–45
D9	GP	Male	X	Public	Phone	30–45
D10	GP	Female	X	Public	Phone	30–45
G11	Gynecology	Female	X	Public	Phone	30–45
G12	Gynecology	Female		Private	Phone	30–45
G13	Gynecology	Male		Private	Phone	30–45

Source: proposed by the authors.

**Table 2:** Physicians’ experience in the field of women’s health.

Years of Experience	Number of Doctors
Less than 10 years	2
Between 10 to 20 years	2
Between 20 to 30 years	1
Between 30 to 40 years	4
More than 40 years	4

Source: proposed by the authors.

**Table 3:** Main questions from the interview guide.

Pre-Established Dimensions	Main Questions
Clinical diseases	Can you tell me how long is your experience as a doctor? What are the main symptoms of infertility diseases? What is the main treatment of infertility diseases?
onsequences of diseases	Do you feel that these diseases are stigmatized? How is the daily life of those women? How hard it is to make the diagnostic of these diseases? What support you have from the government to address these diseases?

Source: proposed by the authors.

**Table 4:** Themes, subthemes and codes.

Themes	Subthemes	Codes
Clinical diseases	Main symptoms of diseases	Painful menstrual periods and intercourses, metabolism problems, excess male hormone, endometrial tissue growth, severe pain, tightening muscles
	Main treatment of diseases	Combined medications, psychological interventions, surgical interventions
Consequences of diseases	For women	Taboo, not being taken seriously, delay/avoid seeking help, infertility, chronic pain, limitations in social and sexual life
	For doctor	Avoid responsibility for diagnosis, erroneous treatments, wrong financing, specialized centers, waiting list of patients and doctors

Source: proposed by the authors.

## References

[1] Langer A., Meleis A., Knaul F.M., Atun R., Aran M., Arreola-Ornelas H., Bhutta Z.A., Binagwaho A., Bonita R., Caglia J.M. (2015). Women and Health: The key for sustainable development. Lancet Commun..

[2] Morice P., Josset P., Chapron C., Dubuisson J.B. (1995). History of infertility. Hum. Reprod. Update.

[3] Zegers-Hochschild F., Adamson G.D., De Mouzon J., Ishihara O., Mansour R., Nygren K., Sullivan E., Van der Poel S., International Committee for Monitoring Assisted Reproductive Technology, World Health Organization (2009). International Committee for Monitoring Assisted Reproductive Technology (ICMART) and the World Health Organization (WHO) revised glossary on ART terminology. Hum. Reprod..

[4] World Health Organization (1992). Recent Advances in Medically Assisted Conception. https://apps.who.int/iris/bitstream/handle/10665/38679/WHO_TRS_820_eng.pdf?sequence=1&isAllowed=y.

[5] Olmedo S., Chillik C., Kopelman S. (2000). Definition and causes of infertility. Reprod. BioMed Online.

[6] Deshpande P., Gupta A. (2019). Causes and Prevalence of Factors Causing Infertility in a Public Health Facility. J. Hum. Reprod. Sci..

[7] Khizroeva J., Nalli C., Bitsadze V., Lojacono A., Zatti S., Andreoli L., Tincani A., Shoenfeld Y., Makatsariya A. (2020). Infertility in women with systemic autoimmune diseases. Best Pract. Res. Clin. Endocrinol. Metab..

[8] Boivin J., Bunting L., Collins J., Nygren K. (2007). International estimates of infertility prevalence and treatment-seeking: Potential need and demand for infertility medical care. Hum. Reprod..

[9] Steiner M., Dunn E., Born L. (2003). Hormones and mood: From menarche to menopause and beyond. J. Affect. Disord..

[10] Roupa Z., Polikandrioti M., Sotiropoulou P., Faros E., Koulouri A., Wozniak G., Gourni M. (2009). Causes of infertility in women at reproductive age. HSJ Health Sci. J..

[11] Cwikela J., Gidronb Y., Sheinerc E. (2004). Psychological interactions with infertility among women. Eur. J. Obstet. Gynecol. Reprod. Biol..

[12] Cousineau T., Domar A. (2007). Psychological impact of infertility. Best Pract. Res. Clin. Endocrinol. Metab..

[13] Winkelman W., Katz P., Smith J., Rowen T. (2016). Sexual Impact of Infertility among Women. Sex. Med..

[14] Sternke E., Abrahamson K. (2015). Perceptions of Women with Infertility on Stigma and Disability. Sex. Disabil..

[15] Greil A. (1991). Not Yet Pregnant: Infertile Couples in Contemporary America.

[16] Moghadam Z., Fereidooni1 B., Saffari M., Montazeri A. (2018). Polycystic ovary syndrome and its impact on Iranian women’s quality of life: A population-based study. Women’s Health.

[17] Lustyk M., Gerrish W., Shaver S., Keys S. (2009). Cognitive-behavioral therapy for premenstrual syndrome and premenstrual dysphoric disorder: A systematic review. Arch. Women’s Ment. Health.

[18] Agarwal S.K., Foster W.D., Groessl E.J. (2019). Rethinking endometriosis care: Applying the chronic care model via a multidisciplinary program for the care of women with endometriosis. Int. J. Women’s Health.

[19] Beck-Peccoz P., Persani L. (2006). Premature ovarian failure. Orphanet. J. Rare Dis..

[20] Cox K.J., Neville C.E. (2012). Assessment and Management Options for Women with Vulvodynia. J. Midwifery Women’s Health.

[21] Apte G., Nelson P., Brisme’e J., Dedrick G., Justiz R., Sizer P. (2012). Chronic Female Pelvic Pain—Part 1: Clinical Pathoanatomy and Examination of the Pelvic Region. Pain Pract..

[22] The World Health Report 2000 from World Health Organization. https://www.who.int/whr/2000/en/whr00_en.pdf?ua=1.

[23] (2018). Euro Health Consumer Index. https://healthpowerhouse.com/media/EHCI-2018/EHCI-2018-report.pdf.

[24] Prime Minister Speech. https://www.dailymail.co.uk/news/article-6603263/Nordic-countries-desperate-babies-Falling-birth-rates-end-welfare-state-model.html.

[25] Prime Minister Speech. https://healthpowerhouse.com/media/EHCI-2018/EHCI-2018-report.pdf.

[26] Prime Minister Speech. https://www.regjeringen.no/en/dokumenter/meld.-st.-18-20182019/id2639253/sec1.

[27] Statistics Norway. https://www.ssb.no/en/statbank/table/04232/.

[28] Olsen J., Zhu J.L., Ramlau-Hansen C.H. (2011). Has fertility declined in recent decades?. Acta Obstetricia et Gynecologica Scandinavica.

[29] Miles M., Huberman A., Saldaña J. (2019). Qualitative Data Analysis.

[30] Mason J. (2018). Qualitative Researching.

[31] Patton M. (2014). Qualitative Evaluation and Research Methods.

[32] Greil A. (1997). Infertility and psychological distress: A critical review of the literature. Soc. Sci. Med..

[33] Taghipour A., Karimi F., Roudsari R., Kimiaei S., Mazlom S., Amirian M. (2017). Women’s perceptions and experiences of the challenges in the process of male infertility treatment: A qualitative study. Electron. Phys..

[34] Remes O., Whitten A., Sabarre K., Phillips K. (2012). University students’ perceptions of environmental risks to infertility. Sex. Health.

[35] Hassanzadeh-Bashtian M., Khadivzadeh T., Badiee-Aval S., Esmaily H. (2018). The Perception and Experience of Infertile Women Who Received Acupressure in Relation to Anxiety: A Qualitative Study. Iran. J. Nurs. Midwifery Res..

[36] Morse J.M. (2015). Data were saturated. Qual. Health Res..

[37] Bowling A. (2002). Research methods in health. Investigating Health and Health Services.

[38] Acker J., Barry K., Essevelde J. (1983). Objectivity and truth: Problems in doing feminist research. Women Stud. Int. Forum.

[39] Popping R. (2000). Computer-Assisted Text Analysis.

[40] Teede H.J., Misso M.L., Boyle J.A., Garad R.M., McAllister V., Downes L., Gibson-Helm M., Hart R.J., Rombauts L., Moran L. (2018). Translation and implementation of the Australian-led PCOS guideline: Clinical summary and translation resources from the International Evidence-based Guideline for the Assessment and Management of Polycystic Ovary Syndrome. Med. J. Aust..

[41] Teede H., Misso M., Costello M., Dokras A., Laven J., Moran L., Piltonen T., Norman R. International Evidence-Based Guideline for the Assessment and Management of Polycystic Ovary Syndrome 2018. https://www.monash.edu/__data/assets/pdf_file/0004/1412644/PCOS_Evidence-Based-Guidelines_20181009.pdf.

[42] Bulletti C., Coccia M.E., Battistoni S., Borini A. (2010). Endometriosis and infertility. J. Assist. Reprod. Genet..

[43] Denny E. (2004). Women’s experience of endometriosis. J. Adv. Nurs..

[44] Chavarro J.E., Rich-Edwards J.E., Audrey J., Gaskins A.J., Farland L.V., Terry K.L., Zhang C., Stacey A., Missmer S.A. (2016). Contributions of the Nurses’ Health Studies to Reproductive Health Research. Am. J. Public Health.

[45] Edwards L. (2003). New concepts in vulvodynia. Am. J. Obstet. Gynecol..

[46] Arnold L.D., Bachmann G.A., Rosen R., Rhoads G.G. (2007). Assessment of vulvodynia symptoms in a sample of US women: A prevalence survey with a nested case control study. Am. J. Obstet. Gynecol..

[47] Haefner H.K., Collins M.E., Davis G.D., Edwards L., Foster D.C., Hartmann E.H., Kaufman R.H., Lynch P.J., Margesson L.J., Moyal-Barracco M. (2005). The Vulvodynia Guideline. J. Low Genit. Tract Dis..

[48] Almeida T., Comber R., Balaam M. (2016). HCI and Intimate Care as an Agenda for Change in Women’s Health. HCI Gend..

[49] Lee C., Dobson A.J., Brown W.J., Bryson L., Byles J., Warner-Smith P., Young A.F. (2015). Cohort Profile: The Australian Longitudinal Study on Women’s Health. Int. J. Epidemiol..

[50] Kjemlff K.H., Erickson B.A., Langenberg P.W. (1996). Chronic Gynecological Conditions Reported by US Women: Findings from the National Health Interview Survey, 1984 to 1992. Am. J. Public Health.

[51] Werner A., Malterud K. (2003). It is hard work behaving as a credible patient: Encounters between women with chronic pain and their doctors. Soc. Sci. Med..

[52] Moghadam Z.B., Fereidooni B., Saffari M., Montazeri A. (2018). Measures of health-related quality of life in PCOS women: A systematic review. Int. J. Women’s Health.

[53] Laganà A.S., Rosa V.L., Rapisarda A.M., Valenti G., Sapia F., Chiofalo B., Rossetti D., Frangež H.B., Bokal E.V., Vitale S.G. (2017). Anxiety and depression in patients with endometriosis: Impact and management challenges. Int. J. Women’s Health.

[54] Harlow B.L., Stweward E.G. (2003). A population-based assessment of chronic unexplained vulvar pain: Have we underestimated the prevalence of Vulvodynia?. J. Am. Med. Women’s Assoc..

[55] Hadfield R., Mardon H., Barlow D., Kennedy S. (1996). Delay in the diagnosis of endometriosis: A survey of women from the USA and the UK. Hum. Reprod..

[56] Ballweg M.L. (2004). Impact of endometriosis on women’s health: Comparative historical data show that the earlier the onset, the more severe the disease. Best Tract Res. Clin. Obstret. Gynaecol..

[57] Stall A.H., Zander M., Nap A.W. (2016). Diagnostic delay of endometriosis in the Netherlands. Gynecol. Obstet. Investig..

[58] Sadownik L.A. (2014). Etiology, diagnosis, and clinical management of vulvodynia. Int. J. Women’s Health.

